# Development of novel bacterial cellulose composites for the textile and shoe industry

**DOI:** 10.1111/1751-7915.13387

**Published:** 2019-05-22

**Authors:** Marta Fernandes, Miguel Gama, Fernando Dourado, António Pedro Souto

**Affiliations:** ^1^ 2C2T – Centre for Textile Science and Technology University of Minho Campus de Azurém 4800‐058 Guimarães Portugal; ^2^ CEB – Centre of Biological Engineering University of Minho Campus de Gualtar 4710‐057 Braga Portugal

## Abstract

This research aimed at producing malleable, breathable and water impermeable bacterial cellulose‐based nanocomposites, by impregnating bacterial cellulose (BC) membranes with two commercial hydrophobic polymers used in textile finishing, Persoftal MS (polydimethylsiloxane) and Baygard EFN (perfluorocarbon), by an exhaustion process. These hydrophobic products penetrated the BC membranes and adsorbed tightly onto the surface of the nanofibres, across the entire depth of the material, as demonstrated by Scanning Electron Microscopy and Fourier Transform Infrared spectroscopy studies. The water static contact angles, drop absorption over time and vapour permeability values showed that the composites were impermeable to liquid water but permeable to water vapour. The mechanical properties of the BC‐nanocomposites were improved after incorporation of the hydrophobic products, in some of the formulations tested, overall presenting a satisfactory performance. Thus, through a simple and cost‐effective process, hydrophobized, robust, malleable and breathable nanocomposites based on BC were obtained, featuring promising properties for application in the textile and shoe industries.

## Introduction

Bacterial cellulose (BC) is a 3D nanofibrillar biopolymer produced through fermentation by bacteria such as the genus *Komagataeibacter*. Under static culture conditions, BC is produced as a gelatinous film consisting of a 3D nanofibrillar arrangement of pure cellulosic fibres. Despite the identical chemical composition to that of plant cellulose, BC differs in structure and mechanical properties, presenting several distinct advantages (Lee *et al*., 2009; Wan *et al*., 2009; Wu *et al*., 2016). First, BC is free of lignin, hemicellulose and pectin, which are present in plant cellulose, and therefore, no extra processing is required for purification. At the microstructure level, BC features a unique porous interconnected structure. The particular mechanical properties of BC arise from its’ randomly organized three‐dimensional network of interconnected nanofibres, with a diameter of 20–100 nm and several micrometres in length, resulting in a high specific surface area (Tang *et al*., 2015; Wu *et al*., 2016), properties which are very advantageous for the production of composite materials (Lee *et al*., 2009). These nanofibres can be oriented uniaxially by the application of tension during drying (Lee *et al*., 2014). Regarding physical properties, BC exhibits high crystallinity, which, coupled with its’ 3D nanofibrillar architecture, results in a high Young's modulus. It also has high degree of polymerization, high water holding capacity and high moldability *in situ* (during fermentation) and *ex situ* (after fermentation) (Lee *et al*., 2009). These unique properties have sustained the elevator pitch of several BC applications in the biomedical field, pulp and paper, composites and foods (Andrade *et al*., 2010; Dourado *et al*., 2016; Fortunato *et al*., 2016; Gonçalves *et al*., 2016; Padrão *et al*., 2016).

Despite these excellent properties, the loss of flexibility upon drying is a disadvantage for several applications such as in the textile and shoe industry. Due to the collapse of the 3D nanofibrillar BC network, a significant reduction in gas permeability also occurs, heavily reducing the material's breathability. Further, the hydrophilic nature of BC hinders the combination with hydrophobic polymer matrixes, an obstacle to the development of composites where both BC and the added polymer contribute to the final desirable properties. Several studies have been conducted on the chemical modification/hydrophobization of cellulose and of BC in particular, attempting to overcome these issues (Tomita *et al*., 2009; Nisoa and Wanichapichart, 2010; Tomé *et al*., 2010; Wan *et al*., 2017).

The major limitations of *ex situ* BC modification methods concern with the size and nature of the reinforcing materials, namely only soluble polymers and nanosubmicron sized materials can penetrate into the BC 3D network. The bulk distribution of these particles within the BC pellicle is also heterogeneous, a feature further aggravated by the hydrophobic nature of certain polymer matrices, which have poor interfacial adhesion to native BC (Shah *et al*., 2013).

For several decades, the development of leather analogues has been pursued by the scientific community and shoe industry. This effort led to the appearance of various materials, both synthetic and natural. Despite the increasing interest and market pull, the commercial penetration of these alternative products has been relatively modest, due to high production costs, low breathability, high stiffness, accelerated discoloration, among other limitations. Also, recent market trends towards the identification of natural non‐cotton derived textiles are emerging. The newly developed strategy here presented, based on BC, aims at meeting the market pull from both the shoe and textile industries regarding the need for new high performance natural materials.

Throughout this research, a novel approach was tested for the bulk and surface modification of BC, combining simplicity, potential for application at large scale and low cost, based on the use of an exhaustion process. Through this process, two hydrophobic commercial polymers, Persoftal MS Con.01, a softener based on polydimethylsiloxane (PDMS), and Baygard EFN, a hydrophobizer based on perfluorocarbon (PFC), were incorporated into the nanofibrillar matrix of BC, aiming at obtaining a malleable, breathable and water impermeable nanocomposite with strong potential of application in textile and shoe industries.

## Results and discussion

Simply defined, the exhaustion process used in textile technology involves placing the fabric or yarn in a chamber containing water and treatment products. The chamber is then sealed and the treatment solution heated, which results in the products transitioning from the water to the fabric or yarn. This procedure was adopted in this work to process BC membranes, incorporating different hydrophobic materials into the cellulosic porous network. Although several authors addressed the development of composites using BC fibres, very few papers use the intact membranes obtained by static fermentation. Thus, we believe this approach is innovative and, by preserving the mechanical properties and the three‐dimensional BC membrane, fully exploits its potential as a nanocomposite scaffold.

### Scanning electron microscopy (SEM)

**Figure 1:**
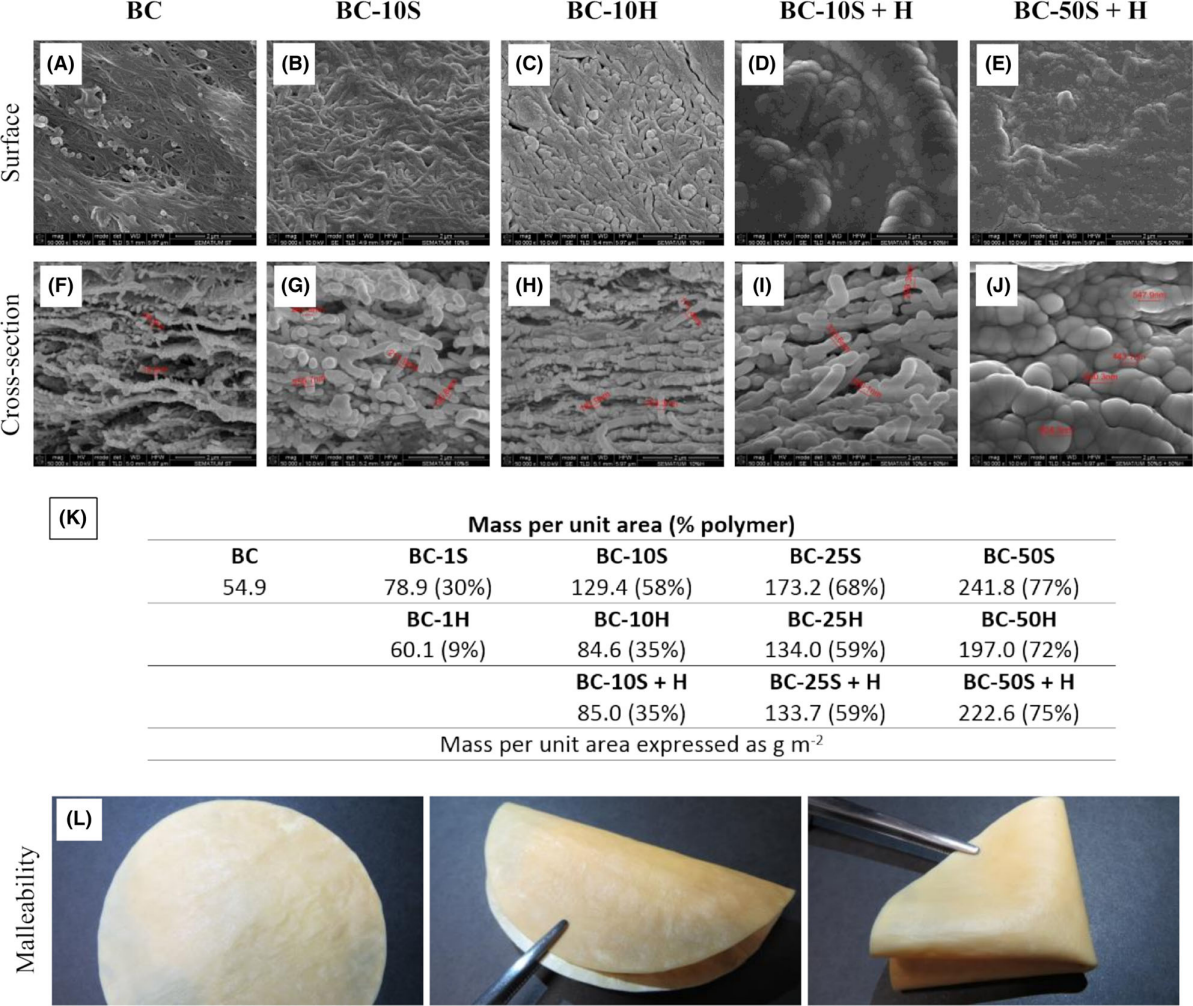
SEM micrographs of BC (A, F) and BC composites (B, G – 10S; C, H – 10H; D, I – 10S + H; and E, J – 50S + H), mass per unit area (K) with percentage of polymers in brackets, and photos showing the malleability (sample BC‐50S + H) (L).

The surface and cross‐section morphologies of the BC and the composites produced with PDMS (**S** – softener) and PFC (**H –** hydrophobizer) polymers (Fig. 1) were observed by SEM analysis. Dried BC (Fig. 1A and F) exhibited a characteristic entangled nanofibrillar matrix disposed in layers with nanofibres of about 70 nm in thickness. Figure 1G (10S) and Fig 1H (10H) shows the increase in the nanofibres thickness, following polymer impregnation. Treating BC with increasing amounts of either **S** or **H** increased the mass per unit area of the BC composite (Fig. 1K), especially with the former, possibly due to a higher penetration of the polymer into the BC matrix and/or higher affinity for the BC's hydroxyl groups. As observed in Fig. 1B, the BC‐10S composite presented thicker nanofibres, with higher surface coverage and a more compact, fused morphology, whereas BC‐10H (Fig. 1C) showed a more porous surface structure. Regarding the sequential impregnation of **S** and **H** (Fig. 1D, E, I, J and M), adding **H** to BC‐S after drying did not impact significantly on the final composite's specific mass, suggesting a limited absorption, as compared to samples treated with **S** alone. Actually, a slight decrease in the composite's mass was observed, as compared with treatments with **S** alone, possibly associated with desorption of **S** during the second stage of the sequential process.

As demonstrated further bellow (regarding the water vapour permeability values, Fig. 6), despite the extensive coverage of the BC nanofibres and compact appearance of the dried composites (Fig. 1), the porosity was not compromised, thus assuring the breathability of the composite. Figure 1L shows photos of the composite 50S + H, demonstrating that it can be easily folded without causing visible damage.

### Atomic force microscopy (AFM)

**Figure 2:**
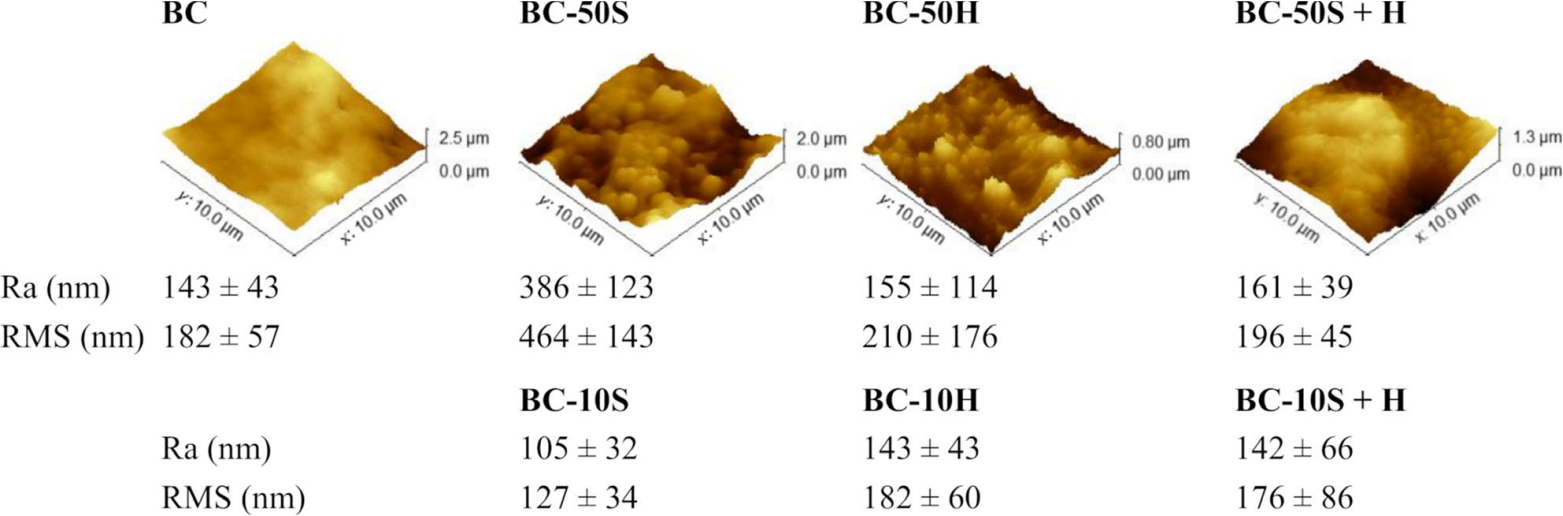
AFM micrographs and results of average roughness (Ra) and root mean square roughness (RMS) of BC and BC composites.

The surface topography of the BC and its composites was also characterized using AFM. Figure 2 illustrates examples of three‐dimensional AFM images of a scanned area of 10 × 10 μm, showing the values of the average roughness (Ra) and the root mean square (RMS) roughness.

The image recorded for the pure BC indicates that the dried membrane has an average surface roughness of 143 nm with a maximum height of 2.5 μm. Following polymer impregnation with 10S, the average roughness was reduced to 105 nm. It may be concluded that the micron‐scale pores of the BC membrane became filled with the polymers, following treatment. In the case of 10H, no effect (within experimental variability) on the surface roughness was observed. These results are in accordance with the SEM observations, as above discussed. In contrast, and associated with the highest increase in the mass per unit area, the addition of higher concentration of **S** increases the most (to 386 nm) the composite's surface roughness. Thereafter, these values decrease with the addition of more polymer **H** (BC‐50S + H). Despite the 2‐fold increase in the mass per unit area (Fig. 1K), increasing the amount of **H** by 5 times (from 10H to 50H) only slightly increases the surface roughness.

It should be emphasized that the surface topography of the BC and BC composites were non‐uniform, as can be noticed by the standard deviation values presented. The results from ANOVA tests show that Ra means difference are not significant at the 0.05 level, and RMS means are statistically different only between 50S with BC, 10S, 10H and 10S+H samples.

### Fourier Transform InfraRed (FTIR)

**Figure 3:**
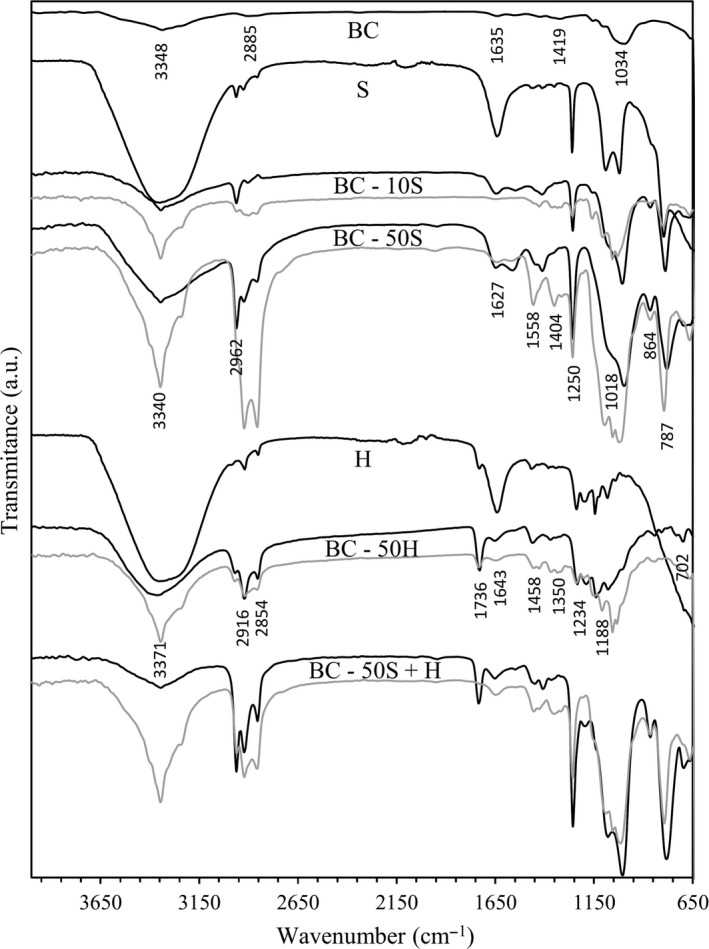
FTIR spectra of BC, PDMS (**S**) and PFC (**H**) based formulations, and BC composites (black lines – exterior; grey lines – interior).

FTIR spectroscopy was used to characterize the composites. As seen in Fig. 3, the spectrum of raw BC presents a band at around 3348 cm^−1^ that corresponds to O–H stretching. The absorption bands at 2985 cm^−1^ and 1419 cm^−1^ are assigned to the CH_2_ and the peak at 1034 cm^−1^ to the vibrations of the glycosidic C–O–C bridges. The band at around 1635 cm^−1^ is assigned to adsorbed water (Garside and Wyeth, 2003; Yang *et al*., 2017).

The main peaks for the samples treated with **S** based product are located at 3340 cm^−1^ (O–H and N–H stretching), 2962 cm^−1^ and 2916 cm^−1^ (asymmetric and symmetric CH_3_ stretching in Si–CH_3_), 1627 cm^−1^ and 1558 cm^−1^ (N–H deformation in amine groups), 1450 cm^−1^ and 1250 cm^−1^ (CH_3_ asymmetric and symmetric bending in Si–CH_3_), 1404 cm^−1^ (CH_2_ bending), 1080 cm^−1^ and 1018 cm^−1^ (asymmetric and symmetric Si–O–Si stretching), 864 cm^−1^ and 787 cm^−1^ (−CH_3_ rocking and Si–C stretching in Si–CH_3_) (Hashem *et al*., 2009; Cai *et al*., 2010; Johnson *et al*., 2013; Zhang *et al*., 2018).

For BC samples treated with **H**, the main peaks observed are located at 3371 cm^−1^ (O–H stretching), 2916 cm^−1^ and 2854 cm^−1^ (C–H asymmetric and symmetric stretching), and at 1458 cm^−1^ (CH_2_ in‐plane bending). The characteristics peaks from the reactive acrylic group which is usually present in short‐chain perfluorinated oligomers are detected at 1736 cm^−1^ (C=O stretching) and 1643 cm^−1^ (C=C stretching). Fluorinated functional groups are identifiable at 1350 cm^−1^ (terminal CF_3_ group vibration), at 1234 cm^−1^ and 1188 cm^−1^ (asymmetric and symmetric stretching vibrations of CF_2_ respectively_)_, and at 702 cm^−1^ (amorphous CF_2_ deformation) (Mukherjee *et al*., 2013; Joseph and Joshi, 2018).

The spectra obtained reveal the presence of the polymers in the interior (bulk) and exterior (surface) of the composites, when softener and hydrophobizer are used alone with BC. However, when combined formulations of **S **+ **H** were used, **H** was not detected inside the composites.

The increase in the BC nanofibrils thickness (as observed by SEM) correlated well with the increase in the infrared absorption peaks’ intensity, as the concentration of the applied products increased. Similar observations were recorded for all other mixtures (data not shown).

### Water contact angle (CA) and surface free energy (SFE)

**Figure 4:**
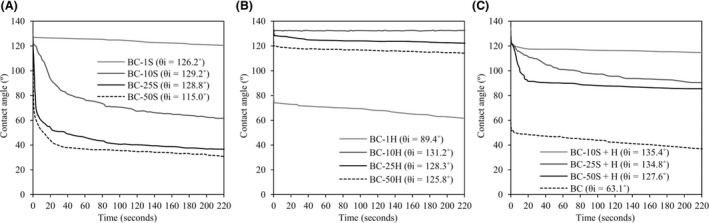
Contact angles over time of the BC composites produced with **S** (A), **H** (B), with **S** and **H** applied sequentially, and BC (C).

Hydrophobic surfaces may be obtained through the creation of hierarchical roughness or through the control of the surface chemistry, decreasing the surface energy (Mondal *et al*., 2018). The introduction of hydrophobic polymers in BC membranes changed its surface wettability, as shown by static contact angle (Table 1) and water drop absorption over time (Fig. 4), as assessed by contact angle (CA) measurements. As expected, the lowest water CA was measured on BC alone (63.8°). After incorporation of the polymers, overall, higher contact angles were obtained, indicative of more hydrophobic surfaces. It is important to mention that, alongside with surface chemistry, surface topography plays a role on wettability. The actual and apparent contact angle values can deviate from each other depending on the surface roughness. Surface hydrophobicity can be enhanced due to surface roughness (Zhao *et al*., 2015). Thus, although slight differences were observed among the samples, these may be related to the surface topography and not just to the surface chemistry. As a matter of fact, the BC fibres are well coated with **S** in the series of samples impregnated with 1% up to 50%, as observed by SEM; thus, the observed differences cannot be assigned to the surface chemistry only.

**Table 1:** Average contact angle values (°) measured for drops of water, PEG 200 and glycerol

Sample	Water	PEG 200	Glycerol
BC	63.8 ± 4.7	38.3 ± 2.3	105.0 ± 4.1
BC‐1S	126.2 ± 1.9	108.1 ± 2.2	126.4 ± 1.1
BC‐10S	129.2 ± 0.5	115.2 ± 3.0	129.4 ± 1.3
BC‐25S	128.4 ± 2.1	108.0 ± 2.8	125.3 ± 1.3
BC‐50S	115.0 ± 4.4	98.4 ± 0.7	112.3 ± 1.5
BC‐1H	89.4 ± 5.2	66.5 ± 1.7	125.7 ± 1.7
BC‐10H	131.2 ± 2.7	101.2 ± 3.8	123.6 ± 2.7
BC‐25H	128.3 ± 0.8	101.1 ± 1.0	127.3 ± 3.8
BC‐50H	125.8 ± 2.5	101.5 ± 1.7	122.4 ± 3.3
BC‐10S + H	135.4 ± 1.0	113.4 ± 1.8	124.7 ± 3.4
BC‐25S + H	134.8 ± 1.0	117.4 ± 1.6	128.3 ± 1.1
BC‐50S + H	127.6 ± 2.5	112.3 ± 0.9	123.7 ± 1.6

Despite the overall proximity in the values of the static contact angles between **S** and **H** composites, the later showed lower permeability to water, as observed from the significantly slower water absorption rates over time (Fig. 4A and B). As a matter of fact, the use of **H** seems to more effectively provide water resistant properties. The reason for this clear trend is not obvious, taking in account the static contact angle or the surface energy (as discussed ahead), and may be related to the ultrastructure, namely porosity, of the composites.

The surface energy is an important variable to understand the wetting phenomena and can be determined from the measurement of the contact angles formed by liquids with different surface energies. Wu's approach (Wu, 1971) was used in this study for monitoring the surface energy and its components on the BC composites.

**Figure 5:**
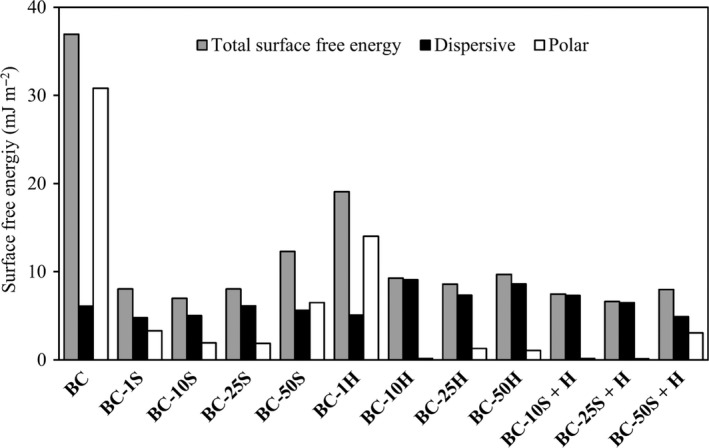
Total surface energy, dispersive and polar components, determined with the Wu method, using the initial contact angles of water, PEG 200 and glycerol.

The polar and dispersive components of the surface free energy of unmodified BC and the BC composites are displayed in Fig. 5. The results show that, overall, comparatively to BC, BC composites obtained by treatment with **S** or **H** and by sequential impregnation (**S **+ **H**) had a noticeable decrease in the surface free energy, due to the reduction of the polar component.

### Water vapour permeability (WVP) and static water absorption (SWA)

Breathability of textiles and leather products is commonly assessed through the measurement of water vapour permeability. This is very important for proper moisture management and to ensure the thermophysiological comfort of the human body. An adequate skin temperature balance must be achieved through perspiration and breathability (Tang *et al*., 2014; Mukhopadhyay *et al*., 2018).

**Figure 6:**
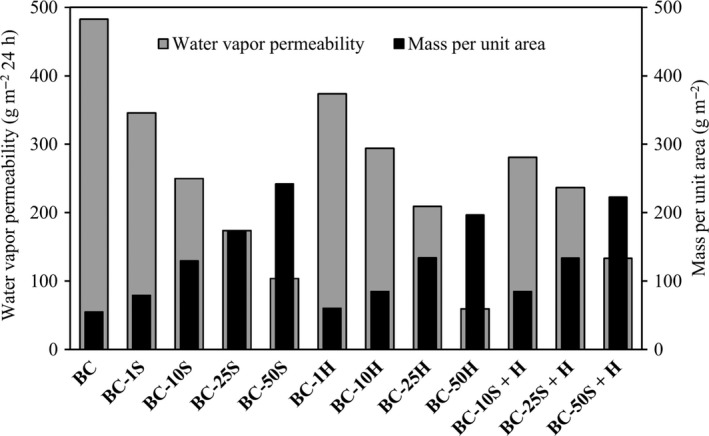
Water vapour permeability and mass per unit area.

As observed in Fig 6, the WVP values of the BC composites decreased when compared to the original dry BC. However, all BC composites were still breathable. Indeed, according to the technical report ISO/TR20879 (ISO, 2007), that establishes the WVP performance requirements for upper footwear components, most of the composites are suitable to be used in footwear (casual footwear – WVP ≥ 192 g m^−2^ 24 h).

Also, with an increase in the amount of incorporated **S** or **H**, (as observed by the increase in the mass per unit area (Fig. 6) and thickness (Table 2)) a decrease in the WVP was observed, as could be expected. In general, the sequential treatment using both polymers did not change significantly the WVP values, as compared to BC samples treated only with the softener.

**Table 2:** Thickness, tensile strength and elongation at break

Sample	Thickness (mm)	Young's modulus (MPa)	Tensile strength (MPa)^a^	Elongation at break (%)
BC	0.30 ± 0.01	9.19 ± 0.30	35.60 ± 3.10	3.44 ± 0.08
BC‐1S	0.34 ± 0.01	18.38 ± 6.15	42.64 ± 7.26	3.45 ± 0.82
BC‐10S	0.48 ± 0.03	6.14 ± 0.78	35.61 ± 2.59	5.10 ± 0.17
BC‐25S	0.63 ± 0.04	1.57 ± 0.28	27.18 ± 1.40	6.90 ± 1.55
BC‐50S	0.73 ± 0.02	1.35 ± 0.50	16.31 ± 0.26	7.67 ± 0.27
BC‐1H	0.30 ± 0.01	23.10 ± 2.37	40.04 ± 10.36	2.82 ± 0.73
BC‐10H	0.34 ± 0.02	11.72 ± 1.56	48.35 ± 8.80	5.92 ± 0.83
BC‐25H	0.50 ± 0.02	3.90 ± 0.21	27.78 ± 0.77	7.97 ± 0.65
BC‐50H	0.61 ± 0.08	3.35 ± 0.85	25.46 ± 2.18	6.04 ± 0.39
BC‐10S + H	0.37 ± 0.02	5.17 ± 0.38	37.25 ± 3.92	5.48 ± 0.29
BC‐25S + H	0.53 ± 0.02	3.37 ± 0.90	31.78 ± 6.71	8.94 ± 0.95
BC‐50S + H	0.77 ± 0.03	2.32 ± 0.62	17.29 ± 0.63	6.37 ± 0.72

First set: 1S/25S; BC/50S; 1S/50S; 10S/50S; 25S/50S; Second set: 10H/25H; 10H/50H; Third set: BC/50S + H; 10S + H/50S + H; 25S + H/50S + H.

a Multiple comparison tests between BC and each set of composites, means difference is significant at the 0.05 level.

The comfort of clothing and footwear is closely associated with both water vapour permeability and static water absorption. In the case of footwear, sweat is first absorbed by the lining and insole material and then the moisture is transferred to the exterior. The materials used inside the shoe should therefore have a good level of water absorption so that the sweat does not accumulate, causing discomfort. The recommended water absorption is a maximum of 60% after 120 min for uppers and a minimum of 100% for linings and insoles (Bitlisli *et al*., 2005).

**Figure 7:**
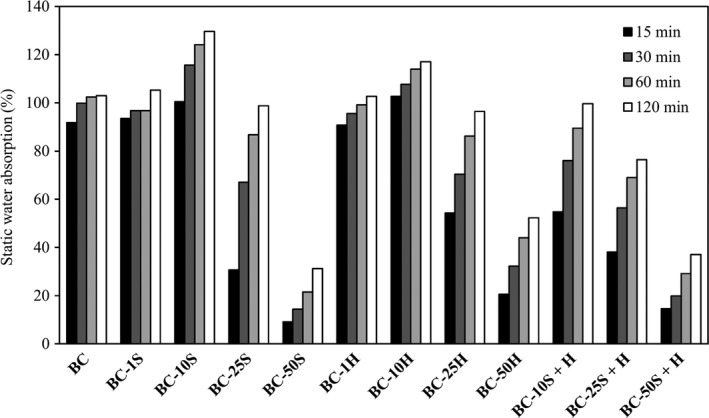
Static water absorption.

The static water absorption was in this work measured at 15, 30, 60 and 120 min. The results are shown in Figure 7. The composites with higher amounts of added polymers (50S, 50H and 50S + H) absorbed less than 60% of water after immersion for 120 min, thus being appropriate for uppers, whereas most of the other composites show an absorption higher than 100% and are more suitable to be used in linings and insoles.

### Mechanical properties

The characterization of the mechanical properties resulting from the tensile test is shown in Table 2. The tensile strength and elongation break of dried BC were found to be of 35.60 MPa and 3.44% respectively. Incorporation of increasing amounts of **S** or **H** increased the thickness and the mass per unit area of the composites, while decreasing the WVP. Unexpectedly, the mechanical properties of the composite did not follow this progression. Regarding the incorporation of softener, maximum tensile strength values were of 42.64 MPa (1S), whereas the maximum elongation at break observed was 7.67% (50S). As for the hydrophobizer, the maximum values of tensile strength and elongation at break were observed, respectively, for 10% and 25% of polymer impregnation. As observed by SEM, the BC composites with lower amounts of polymer absorbed formed thicker fibres. Additionally, the high interfacial interaction between the added polymers and the cellulose nanofibres through hydrogen bonding allows an efficient distribution of the stress, resulting in an improvement of the mechanical properties. However, as the concentration of the added products increase, the tensile strength and Young modulus start to decrease. This observation can be explained by the complete coating of surface hydroxyl groups of the cellulose nanofibres, preventing their contribution to the mechanical strength and elasticity through H‐bonding. As previously reported (Soykeabkaew *et al*., 2009; Gea *et al*., 2010; Asgher *et al*., 2017), the strong attractive hydrogen bonding between cellulose nanofibres tends to weaken owing to extensive surface coating, affecting negatively the mechanical properties. In our composites, the quantity of BC (reinforcement) is constant. As more polymers (matrix) are incorporated, the relative percentage of the reinforcement decreases, the behaviour of the composite becoming increasingly governed by the matrix. Therefore, the mechanical strength and elasticity of the composite becomes gradually dependent on the intermolecular bonding of the matrix polymers. In the case of the softener polydimethylsiloxane, the weak intermolecular forces between the methyl groups yield very low tensile strength (Paquien *et al*., 2005; Jin *et al*., 2018). Also, fluorine atoms have low polarizability that results in low surface energies and thus weak cohesive forces between fluorocarbon molecules (Lemal, 2004).

It is also observed that samples with higher amounts of incorporated polymers have higher elongation, due to the greater mobility allowed between the different layers of the BC membrane.

Finally, no significant differences were achieved by sequentially incorporating **H** in samples pretreated with **S**. Overall, taking as reference the technical report ISO/TR20879 (ISO, 2007), that establishes the performance requirements for uppers components for footwear, the composites present suitable mechanical properties [casual footwear – Breaking strength ≥ 10 N mm^−1^, elongation ≥ 7% (along)]. Regarding tensile strength, all samples are above or near the reference value (MPa × Thickness), although regarding the elongation at break only 50S, 25H and 25S + H samples match the requirements.

In this study, through an exhaustion process, BC fibres were surface modified with Persoftal MS Con.01 and Baygard EFN, resulting in malleable and mechanically resistant composites, with hydrophobic character and breathability. The new BC‐based composites may offer a sustainable alternative to cotton, leather and man‐made cellulosic fibres, thus exhibiting strong potential for further high value‐added differentiation and sustainable consumer products such as textiles and leather. BC‐based composites may be regarded as strategic materials with enormous potential, focusing on natural and organic products development and represent an alternative to materials used today in textile and shoe industries.

## Experimental procedures

### Materials

BC membranes were offered by Satisfibre S.A. (Portugal). The commercial polymer formulations, Persoftal MS Conc.01 (a softener based on Polydimethylsiloxane – PDMS) and Baygard EFN (hydrophobizer based on Perfluorocarbon – PFC), both from Tanatex Chemicals, were offered by ADI Center Portugal.

Perfluorinated acrylate polymers have extremely low surface energy due to their side chain containing outwardly oriented perfluoro hydrophobic groups and are widely used in textile and leather coatings. The application of perfluorinated organic compounds with a long perfluoroalkyl chain (C_*n*_F2_*n*+1_, *n* ≥ 8) has been restricted by the European Union due to its high bioaccumulation and difficult biodegradation (Zahid *et al*., 2017). Thus, the hydrophobic product used in this work consisted of a C6‐based Fluorocarbon polymer nano‐emulsion without perfluorooctane sulfonate (PFOS) and less than 5 ppb perfluorooctanoic acid (PFOA). The characteristics of the products used are summarized in Table 3.

**Table 3:** Characteristics of the finishing polymers used.^a^

	Softener (S) – Persoftal MS Conc.01	Hydrophobizer (H) – Baygard EFN
Chemical basis	Modified polysiloxane aqueous dispersion	Fluorocarbon polymer aqueous nano‐emulsion
Ionicity	Non‐ionic	Non‐ionic
Density	0.99 g cm^−3^ (23°C)	1.1 g cm^−3^ (20°C)
Viscosity	405 mPa s (23°C)	100 mPa s (20°C)
Content	10–20% Siloxanes and Silicones^b^ (CAS: 75718‐16‐0)	28% Fluorocarbon polymer;
	3–5% Alcohols, C12‐18, ethoxylated (CAS: 68213‐23‐0)	0.1‐1% Alcohols, C16‐20, ethoxylated (CAS: 106232‐82‐0)
	3–5% Alcohols, C10‐14, ethoxylated (CAS: 66455‐15‐0)	
	0.1–1% Octamethylcyclotetrasiloxane (CAS: 556‐67‐2)	

a Information extracted from the technical sheets provided by the manufacturer.

b 3‐((2‐aminoethyl)amino)propyl Me, diMe, hydroxy‐terminated.

### Composites production

BC membranes (with about 2.5–3.0 cm in thickness, with a size of 12.0 × 13.0 cm and weighting 450 g) were squeezed to a final wet mass of 100 g (2.6% BC (w/w)). The compressed membranes were each treated by exhaustion in 100 ml of:

- an aqueous mixture containing either the softener (**S**) or the hydrophobizer (**H**), each, at different concentrations (1, 10, 25 and 50%, v/v), or;
- processed in two steps by exhaustion with 10, 25 and 50% (v/v) of softener **S**, followed by drying; afterwards, the dried composites were each impregnated with 50% (v/v) hydrophobizer **H** (further designated by 10S + H, 25S + H and 50S + H).

It is expected that with the sequential application **S **+ **H** a material with suitable properties for application in the textile and shoe industry can be achieved. Considering PDMS characteristics, it allows properties such as malleability, robustness and softness, permitting water vapour transmission. However, since the hydroxyl and amino‐functional polar groups linked to PDMS in the softener used impart some hydrophilicity, the subsequent application of the PFC nano‐emulsion will hydrophobize the surface.

The exhaustion process was carried out in an Ibelus machine equipped with an infrared heating system, using stainless steel cups with a capacity of approximately 220 cm^3^, with a rotation of 50 rpm and 40 cycles. The desired temperature (30°C) was achieved using a gradient of 2°C min^−1^. The treatment lasted for 5 days at 30°C, after which the samples were oven dried (WTC binder oven) at 25°C for 48 h, followed by a curing step for 30 min at 120°C. To avoid shrinkage of the samples during drying and curing, the composite BC membranes were attached to a zinc‐plated wire support.

### Physical–chemical properties evaluation

#### Scanning Electron Microscopy (SEM)

BC composites were coated with a thin layer of gold–palladium. Analyses of the surface morphology and cross section of these composites were done using an ultra‐high‐resolution field emission gun SEM instrument (NOVA 200 Nano SEM; FEI, Hillsboro, OR, USA).

#### Atomic Force Microscopy (AFM)

Atomic force microscope images of the BC composites were collected in tapping mode using a Nanoscope III microscope from Digital Instruments (Santa Barbara, CA, USA), with antimony‐doped silicon probes in contact mode in air. Images were captured at a scanning rate of 1.0 Hz and resolution of 512 pixels × 512 pixels. The roughness parameters, roughness average (Ra) and root mean square (RMS), were calculated using the average of three scans in different places for each sample in 10 μm × 10 μm area.

#### FTIR analysis

A Nicolet Avatar 360 FTIR spectrophotometer (Madison, WI, USA) was used to record the FTIR spectra of the BC sheet and BC composites. The spectra were collected in the attenuated total reflection mode (ATR) at a spectral resolution of 16 cm^−1^, with 60 scans, over the range 400–4000 cm^−1^ at room temperature. A background scan with no sample and no pressure was acquired before the spectra of the samples were collected.

#### Surface energy

Contact angles measurements were carried out in a Dataphysics instrument (Filderstadt, Germany) using oca20 software (Germany) with a video system for the capture of images in static mode using the sessile drop method. A drop of 5 μl of distilled water was placed on the composite's surface with a microlitre syringe and observed with a special charge‐coupled device camera. After a water drop was deposited in the composites surface, the water contact angle was observed over time for 220 s. At least five measurements at different places were taken for each sample. The camera recorded an image every 0.04 s. To calculate the surface energy (γ_s_) of the BC composites and their polar (γ_s_^P^) and dispersive (γ_s_^D^) components, the Wu method (harmonic‐mean) was used, with the Eq. (1) (Wu, 1971):

(1) $$\gamma_{\mathrm{sl}}=\gamma_{s}+\gamma_{l}-4\left[\frac{\gamma_{s}^{D}\gamma_{l}^{D}}{\gamma_{s}^{D}+\gamma_{l}^{D}}+\frac{\gamma_{s}^{P}\gamma_{l}^{P}}{\gamma_{s}^{P}+\gamma_{l}^{P}}\right]$$

The following liquids (l) with known surface energy and surface energy components were used: distilled water (γ: 72.8; γ^D^: 29.1; γ^P^: 43.7); polyethylene glycol 200 (γ: 43.5; γ^D^: 29.9; γ^P^: 13.6); and glycerol (γ: 63.4; γ^D^: 37.4; γ^P^: 26.0), units in mJ m^−2^ (Oliveira *et al*., 2013).

#### Water vapour permeability (WVP) and static water absorption (SWA)

WVP was evaluated according to the Standard BS 7209:1990 (British Standards Institution, 1990). According to this method, the test specimen is sealed over the top of a test dish which contains 46 ml of distilled water and the assembly is placed on a rotating turntable and allowed to rotate, under isothermal conditions, for a period of one hour to establish the equilibrium of water vapour pressure gradient across the sample. After this period, the assembly is weighted and allowed to rotate for 24 h and is weighted again.

The result is represented by the following Eq. (2) and expressed in g m^−2^ 24 h.

(2) $$\text{WVP}=\frac{24W}{\mathrm{AT}}$$

where *W* is the mass (g) of water vapour lost in *T* hours, *A* is the area of the sample exposed to vapour (m^2^), and *T* is the time between the various weightings (h).

SWA was measured by immersing the samples in distilled water, at room temperature, for 15, 30, 60 and 120 min. After the immersion time, the specimens were removed from the container and their surface moisture was removed by tissue paper. The content of water absorbed was weighed, and the SWA was calculated through Eq. (3).

(3) $$\text{SWA}=\frac{Mt-M0}{M0}$$

where *Mt* is the weight of specimen at a given immersion time and *M*0 is the weight of the dried sample.

#### Tensile strength

The overall width of the sample (25 mm) was fixed, and a length that allows an initial distance between the clamps of the strength tester equipment (Hounsfield HSK100) of 75 mm was set out between grips; the samples were then submitted to tensile and tear. Three samples of each material were tested at a constant speed of 100 mm min^−1^.

### Statistical analysis

Statistical analysis was performed using origin pro 8 software. All data are presented as the mean ± standard error of the mean. Statistical analysis included Shapiro–Wilks, Skewness and one‐way ANOVA with Tukey's post hoc tests whenever the results displayed a parametric distribution, otherwise Dunn–Sidak post hoc test was used.

## Conflict of interest

None declared.
